# Electrical Stimulation Counteracts Muscle Decline in Seniors

**DOI:** 10.3389/fnagi.2014.00189

**Published:** 2014-07-24

**Authors:** Helmut Kern, Laura Barberi, Stefan Löfler, Simona Sbardella, Samantha Burggraf, Hannah Fruhmann, Ugo Carraro, Simone Mosole, Nejc Sarabon, Michael Vogelauer, Winfried Mayr, Matthias Krenn, Jan Cvecka, Vanina Romanello, Laura Pietrangelo, Feliciano Protasi, Marco Sandri, Sandra Zampieri, Antonio Musaro

**Affiliations:** ^1^Institute of Physical Medicine and Rehabilitation, Wilhelminenspital, Vienna, Austria; ^2^Ludwig Boltzmann Institute of Electrical Stimulation and Physical Rehabilitation, Vienna, Austria; ^3^DAHFMO-Unit of Histology and Medical Embryology, Institute Pasteur Cenci-Bolognetti, IIM, Sapienza University of Rome, Rome, Italy; ^4^Laboratory of Translation Myology, Department of Biomedical Sciences, University of Padova, Padova, Italy; ^5^Science and Research Centre, Institute for Kinesiology Research, University of Primorska, Koper, Slovenia; ^6^Center of Medical Physics and Biomedical Engineering, Medical University of Vienna, Vienna, Austria; ^7^Faculty of Physical Education and Sport, Comenius University, Bratislava, Slovakia; ^8^Dulbecco Telethon Institute at Venetian Institute of Molecular Medicine, Padova, Italy; ^9^CeSI-Center for Research on Aging & DNICS – Department of Neuroscience, Imaging and Clinical Sciences, University G. d’Annunzio of Chieti, Chieti, Italy; ^10^Center for Life Nano Science@Sapienza, Istituto Italiano di Tecnologia, Rome, Italy

**Keywords:** electrical stimulation, aging, muscle performance, muscle atrophy, IGF-1, extracellular matrix, satellite cells, microRNA

## Abstract

The loss in muscle mass coupled with a decrease in specific force and shift in fiber composition are hallmarks of aging. Training and regular exercise attenuate the signs of sarcopenia. However, pathologic conditions limit the ability to perform physical exercise. We addressed whether electrical stimulation (ES) is an alternative intervention to improve muscle recovery and defined the molecular mechanism associated with improvement in muscle structure and function. We analyzed, at functional, structural, and molecular level, the effects of ES training on healthy seniors with normal life style, without routine sport activity. ES was able to improve muscle torque and functional performances of seniors and increased the size of fast muscle fibers. At molecular level, ES induced up-regulation of IGF-1 and modulation of MuRF-1, a muscle-specific atrophy-related gene. ES also induced up-regulation of relevant markers of differentiating satellite cells and of extracellular matrix remodeling, which might guarantee shape and mechanical forces of trained skeletal muscle as well as maintenance of satellite cell function, reducing fibrosis. Our data provide evidence that ES is a safe method to counteract muscle decline associated with aging.

## Introduction

There is considerable clinical interest in therapeutic strategies to counteract muscle wasting associated with aging.

Skeletal muscle is particularly susceptible to the effects of aging, undergoing a steady reduction in function and losing up to a third of its mass and strength. This decline in functional performance is due to an overall decrease in muscle integrity, as fibrosis and fat accumulation replace functional contractile tissue, and to loss of the fastest most powerful fibers (Scicchitano et al., 2009; Vinciguerra et al., 2010).

At present, it is clear that the most efficient method that has been used to counteract age-related muscle weakness is long term physical exercise (Paffenbarger et al., 1994). Physical exercise increases protein synthesis, turnover and satellite cell number, stimulates appetite, increases IGF-1 expression levels, and capillary bed density. We recently reported that physical exercise in seniors preserves muscle morphology and ultrastructure, guarantees a greater maximal isometric force and function, and modulates the expression of genes related to autophagy and reactive oxygen species detoxification (Mosole et al., 2014; Zampieri et al., 2014). Nevertheless, certain pathologic conditions and aging limit the effectiveness of exercise and, therefore, the benefits from it.

An alternative effective intervention to improve muscle recovery is electrical stimulation (ES) (Quittan et al., 2001; Nuhr et al., 2004; Bax et al., 2005; Strasser et al., 2009). ES has been used in clinical settings for rehabilitation purposes, as an alternative therapeutic approach to counteract neuromuscular disability, as well as for muscle strengthening and maintenance of muscle mass in seniors (Maddocks et al., 2013). In addition, there are studies showing that patients with knee osteoarthritis can benefit from the use of ES alone or as an adjunct therapy (Rosemffet et al., 2004; Levine et al., 2013). ES directly stimulates skeletal muscle protein synthesis rates (Wall et al., 2012). Although controversial results have been published as consequence of varying protocols (e.g., training 3–7 times a week, training period from 3–12 weeks) and stimulation parameters (e.g., stimulation duration 2–30 s, stimulation frequency 8–80 Hz) (Giggins et al., 2012; de Oliveira Melo et al., 2013), ES represents a promising adjuvant treatment to attenuate muscle disability. Nevertheless, the molecular mechanisms by which ES exerts its specific anabolic effects on skeletal muscle remain to be elucidated.

Based on our documented clinical experience on the use of ES to rescue permanently denervated skeletal muscles in paraplegics (Kern et al., 2004, 2008, 2010; Ashley et al., 2007; Boncompagni et al., 2007), we verified whether ES can be proposed as a therapeutic tool to rehabilitate skeletal muscle of sedentary seniors.

We demonstrated that ES mimics the beneficial effects of physical exercise in muscle of aging individuals and we defined the molecular signature underlying these effects.

## Materials and Methods

### Subjects enrolled in the study

Sixteen subjects (eight male and eight female) (73.1 ± 6.9 years, 81.7 ± 14.7 kg, 170.3 ± 11.2 cm) were recruited for the study. All of the subjects were volunteers who signed an informed consent and received detailed information about the functional test protocols, the trainings, and muscle biopsies. Approval from the national committee for medical ethics was obtained at the beginning of the study (EK08-102-0608). All subjects included were healthy and declared not to have any specific physical/disease issue and were instructed to maintain their normal daily activities during the training period. Various functional tests, force measurement, and muscle biopsy were performed twice, namely 1 week before and 1 week after 9 weeks of ES training.

### Electrical stimulation training

Subjects were exposed to regular neuromuscular ES training (swelling current) for a period of 9 weeks, starting two times a week for the first 3 weeks and then switched to three times a week for the next 6 weeks, amounting to a total of 24 training sessions (3 × 10 min each session). ES training was performed with a two channel custom-built battery-powered stimulator (Krenn et al., 2011) at home by the subjects themselves after detailed instructions. The subjects applied two conductive rubber electrodes (9 cm × 14 cm; 126 cm^2^), which were attached to the skin by wet sponge on the anterior thigh on both sides (left/right). The electrode pairs for left and right thigh were connected to the two channels of the stimulator. This allowed independent activation of the left and right thigh muscles, which were stimulated in an alternative manner. Each repetition (i.e., ES evoked muscle contraction) was evoked by a 3.5 s train (60 Hz) of electrical pulses (rectangular, biphasic, width 0.6 ms). Consecutive contractions of the same thigh were separated by 4.5 s intervals. In this study, constant voltage stimulation devices were applied. The subjects were instructed to increase the stimulation intensity until their maximum sensory tolerance level was reached. With this intensity all of the subjects achieved full knee extension. Nevertheless, the applied current and voltage was recorded by the stimulation device for each training session. The mean stimulation current was 128 ± 16 mA and voltage of 39 ± 14 V.

### Force measurement

An isometric measurement on a dynamometer (S2P Ltd., Lubljana, Slovenia) as described (Šarabon et al., 2013a,b) with 90° hip flexion and 60° knee flexion (full knee extension = 0°) was performed three times at each leg to assess the maximal isometric torque of the left and right knee extensors. The mean of the best values of each leg were taken for further analyses.

### Functional tests

A complete set of functional tests to access mobility and function in activities of daily living (ADL) was designed and applied to each of the subjects. These tests included: time up and go test (TUGT) (Podsiadlo and Richardson, 1991) where the subjects were asked to stand up from a standard chair, walk a distance of 3 m, turn around, walk back to the chair, and sit down again all as fast as possible; short physical performance battery (SPPB) (Guralnik et al., 1994) to evaluate the lower extremities function by using tests of gait speed (2.4 m), standing balance (side-by-side, semi-tandem, and tandem stance for 10 s) and the time which the subject needed to rise from a chair for five times as quickly as possible with the arms folded across their chest; 12 flight Stair Test (Suzuki et al., 2001) where the participant was instructed to ascend and descend the stairs after reaching the top (12th) step as quick and safe as possible; and 10 m-walking test with habitual and fastest walking speed (but not running) (Šarabon et al., 2010a,b) where each speed was performed three times, the time was measured and average velocity calculated.

### Muscle biopsies

Muscle biopsies were harvested as described (Kern et al., 2004) from the vastus lateralis muscle 15–20 cm proximal of the joint space of the knee, with the Bergström needle inserted perpendicular to the fiber direction. The biopsies before training were taken 10 days after the initial assessment at inclusion to the study, ES training started 14 days later. Post-training biopsies were taken 7 days after the last training session. The final functional assessment was done 4 days after the last training session. About 50–70 mg of tissue was harvested from both legs of the subjects.

#### Histological analysis

For light microscopy analyses, serial cryosections (8 μ thick) from frozen muscle biopsies were mounted on polysine™ glass slides, air-dried, and stained either with Hematoxylin–Eosin (HE) or for myofibrillar ATPases to evaluate muscle fiber type using conventional techniques as described (Rossini et al., 2002). Slow-type muscle fibers are dark-stained, while the fast-type fibers are light-stained following pre-incubation at pH 4.35.

#### Morphometric analysis

The mean myofiber diameter and the percentage of slow and fast-type muscle fibers were evaluated from stained cross sections in accordance with our previous published methods (Rossini et al., 2002; Carraro et al., 2005; Ashley et al., 2007; Biral et al., 2008; Kern et al., 2008, 2010). Images were acquired using a Zeiss microscope connected to a Leica DC300F camera. Morphometry analysis was performed using Scion Image software (2000 Scion Corporation, Inc.).

### Immunofluorescence analysis

Muscle sections were incubated either for 1 h at room temperature (RT) or overnight at 4°C, with anti-neural adhesion molecule (N-CAM) rabbit polyclonal antibody (Chemicon, Italy), anti-Pax7 mouse monoclonal antibody (DSHB, Iowa), or anti-laminin rabbit polyclonal antibody (Sigma, Italy) 1:100 diluted in PBS, respectively, as described (Zampieri et al., 2010; Mosole et al., 2014). Sections were then incubated for 1 h at RT with Cy3 or Alexa Fluor^®^ 488 dye conjugated antibodies against rabbit (Chemicon, Italy) or mouse IgG (Life technologies, Italy). Sections were then mounted on glass slides using ProLong Gold antifade reagent with DAPI (Life Technologies). Quantitation of Pax7 positive cells were performed on captured images from random fields counting a minimum of 300 fibers per biopsy.

### Gene expression analyses and miRNA

Total RNA extraction from human muscle biopsies before and after ES was performed with tissue lyser (Qiagen) in TriRiagentTM (Sigma) and small RNAs were purified using PureLink miRNA Isolation Kit (Invitrogen). This RNA fraction, containing microRNA (miRNA), was reverse-transcribed using the TaqMan^®^ MicroRNA Reverse Transcription Kit (Life Technologies); the other RNA fraction, containing mRNA, was reverse-transcribed using a QuantiTect Reverse Transcription kit (Qiagen). The reverse-transcription reactions were performed according to the manufacturers’ instructions. Quantitative PCR was performed on an ABI PRISM 7500 SDS (Applied Biosystems, USA), using pre-made 6-carboxyfluorescein (FAM)-labeled TaqMan assays for GAPDH, IGF-1 Ea, IGF-1 Eb, IGF-1 Ec, IGF-1 pan, Myostatin, Collagen I, III, VI (Applied Biosystems, USA). FAM-labeled TaqMan MicroRNA Assays for miR-1, miR-133a, miR-206, miR-29, and U6 snRNA (Applied Biosystems, USA) were performed as described. Quantitative RT-PCR sample values were normalized to the expression of GAPDH mRNA or U6 snRNA. The relative level for each gene and miRNA was calculated using the 2-DDCt method (Livak and Schmittgen, 2001) and reported as mean fold change in gene expression.

### Statistical analyses

SPSS Statistics software package, version 17.0 was used to evaluate differences between the measurements in parameters of torque, functional tests, muscle morphometry, and molecular data. Normal distribution was obtained with Shapiro–Wilk-Test, the two-tailed paired and unpaired Student’s *t*-test and Wilcoxon-Test were used for normal and not normal distributed variables, respectively. For differences presented in percentage the 95% confidence interval (CI) was calculated. The level of significance was set to *p* < 0.05.

## Results

### Electrical stimulation improves functional performances after 9 weeks of training

To assess mobility, frailty, and risk of falling, behavior analyses in challenging conditions as TUGT and SPPB is recommended (Freiberger et al., 2013; Viana et al., 2013).

With ES training, we improved (i.e., shortened) the *TUGT* time (−16.4% ± 6.1 CI, *p* < 0.0005) and increased the *SPPB Score* (+11.2% ± 6.8 CI, *p* < 0.005) (Table 1), resulting in a greater mobility in seniors recruited for this study.

**Table 1:** **Force measurements and functional tests of seniors with muscle weakness before and after ES training**.

	Pre	Post	Improvement	*t*-test
**ALL (*N* = 16)**				
Torque (Nm/kg)	1.42 ± 0.34	1.51 ± 0.38	6.0 ± 4.9	***p* < 0.05**
TUGT (s)	8.42 ± 1.95	7.04 ± 1.09	-16.4 ± 6.1	***p* < 0.0005**
5×chair rise (s)	13.85 ± 3.33	10.53 ± 3.63	-23.9 ± 8.6	***p* < 0.005^a^**
SPPB score	10.06 ± 1.39	11.19 ± 1.22	11.2 ± 6.8	***p* < 0.005^a^**
Stair test (s)	15.09 ± 3.48	11.90 ± 2.32	-21.1 ± 10.8	***p* < 0.05**
10 m test habitual (m/s)	1.20 ± 0.19	1.26 ± 0.18	5.3 ± 4.6	***p* < 0.05**
10 m test fast (m/s)	1.58 ± 0.28	1.66 ± 0.24	4.9 ± 3.7	***p* < 0.05**
**FEMALE (*N* = 8)**				
Torque (Nm/kg)	1.35 ± 0.32	1.45 ± 0.37	7.5 ± 7.4	**0.058**
TUGT (s)	9.13 ± 1.82	7.58 ± 0.96	-16.9 ± 9.0	***p* < 0.05**
5×chair rise (s)	13.52 ± 3.30	9.01 ± 1.19	-33.3 ± 12.5	***p* < 0.005**
SPPB score	10.13 ± 1.55	11.75 ± 0.46	16.0 ± 12.1	***p* < 0.05^a^**
Stair test (s)	15.26 ± 2.83	11.12 ± 1.70	-27.2 ± 16.6	**0.054**
10 m test habitual (m/s)	1.09 ± 0.16	1.17 ± 0.14	7.4 ± 8.3	**0.117**
10 m test fast (m/s)	1.41 ± 0.15	1.51 ± 0.14	6.5 ± 6.3	**0.075**
**MALE (*N* = 8)**				
Torque (Nm/kg)	1.50 ± 0.36	1.57 ± 0.41	4.5 ± 6.7	**0.208^a^**
TUGT (s)	7.71 ± 1.92	6.49 ± 0.97	-15.8 ± 8.8	***p* < 0.05**
5×chair rise (s)	14.22 ± 3.60	12.28 ± 4.74	-13.7 ± 9.4	***p* < 0.05**
SPPB score	10.0 ± 1.31	10.63 ± 1.51	6.3 ± 3.6	***p* < 0.05**
Stair test (s)	14.92 ± 4.37	12.69 ± 2.77	-15.0 ± 13.1	**0.161**
10 m test habitual (m/s)	1.31 ± 0.16	1.35 ± 0.17	3.5 ± 3.9	**0.125**
10 m test fast (m/s)	1.74 ± 0.29	1.81 ± 0.22	3.5 ± 4.0	**0.173**

*Values are given as mean ± SD; BMI, body mass index; TUGT, timed up and go test; SPPB, short physical performance battery. Improvement values are presented as difference in percentage ±95% confidence interval*.

*^a^Wilcoxon-Test*.

For older adults, the ability to rise from a chair and sit down five times consecutively is a parameter to measure the degree of independence (Corrigan and Bohannon, 2001; Freiberger et al., 2013) and is considered as an index of muscle strength (Bohannon, 1997). Since the test is specific to lower body strength and power, the significant pre-post-test improvement (−23.9% ± 8.6 CI, *p* < 0.005) of the 5× Chair Rise Test indicates a sufficient training effect of ES (Table 1).

The maximum isometric torque, an important factor for gait and physical function and a key factor against sarcopenia (Cruz-Jentoft, 2013) developed by the Quadriceps (+6.0% ± 4.9 CI, *p* < 0.05), was significantly improved by ES training (Table 1).

The ability to climb stairs in a secure and fast manner is an essential eccentric and concentric strength performance of the lower extremities in daily life (Rejeski et al., 1995). The significant decrease of stair test time (−21.1% ± 10.8 CI, *p* < 0.05) in our ES-treated subjects indicates a greater performance and safety for the ADL (Table 1).

Gait speed is relevant to the functioning of seniors in the community and an important predictor for the onset of disability, commonly used by physical therapists and other clinicians (Guralnik et al., 2000; Bohannon and Williams Andrews, 2011) and known as good predictor for frailty (Cruz-Jentoft, 2013; Viana et al., 2013). The significant increase of the 10 m test habitual as well as fastest walking speed (+5.3% ± 4.6 CI, *p* < 0.05 and +4.9% ± 3.7 CI, *p* < 0.05, respectively) supports the functional changes and are good indicators of prevention of frailty and falls (Table 1).

### Electrical stimulation maintains muscle mass and enhances satellite cells activation, promoting muscle adaptation

**Figure 1:**
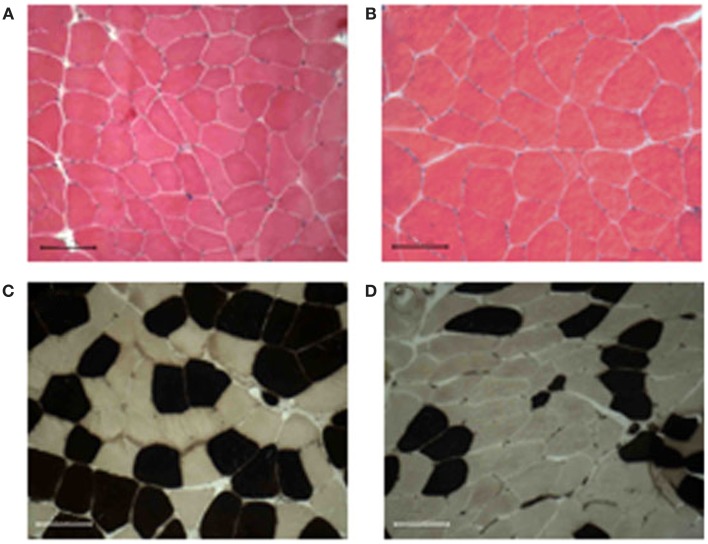
**Muscle morphology and fiber-type distribution**. All muscle biopsies present well-packed myofibers, without signs of fibrosis, and inflammatory cell infiltration before **(A)** or after 9 weeks of training **(B)**. The training induced an increase of either diameter and percentage of the fast-type fibers [brown stained **(C,D)**]. Bar 100 μm.

We also monitored whether the aforementioned functional benefit, exerted by ES, was associated with a morphological gain. ES training maintained the overall mean myofiber diameter (Figures 1A,B; Table 2), while significantly increased the diameter of fast-type myofibers and decreased that of slow fibers type (Figures 1C,D; Table 2). Changes in fiber-type distribution were also observed, even though not significantly (Table 2).

**Table 2:** **Mean myofiber diameter and fiber-type distribution in skeletal muscle biopsies pre- and post-training**.

	Pre	Post	Difference	*t*-test
All fibers
Size (μm)	49.6 ± 15.6	49.5 ± 15.8	−0.3%	N.S
Fast-type fibers
Size (μm)	46.8 ± 14.4	47.8 ± 15.8	+2.2%	***p* < 0.0001**
Percentage	45%	49%	+8.9%	N.S
Slow-type fibers
Size (μm)	50.4 ± 14.8	48.4 ± 16.7	−3.6%	***p* < 0.0001**
Percentage	55%	51%	−7.2%	N.S

*The overall mean myofiber diameter did not significantly change after 9 weeks of training, while a significant increase of fast-type mean myofiber diameter was observed. Values are given in mean ± SD. Bold font indicates statistically significant values. N.S, No statistically significant*.

Of note, no sign of fibrosis and/or inflammatory cell infiltration was detected in treated muscles (Figure 1). Moreover, ultrastructural analysis did not reveal alterations in muscle structure between pre- and post-trained muscles, nor differences in the frequency and position of calcium release units (CRUs) and mitochondria between the two groups of samples (data not shown).

A critical role in muscle homeostasis and regeneration is exerted by satellite cells (Carosio et al., 2011), which can be also activated by different stimuli, including physical exercise (Kadi et al., 1999; Snijders et al., 2009; Walker et al., 2012).

**Figure 2:**
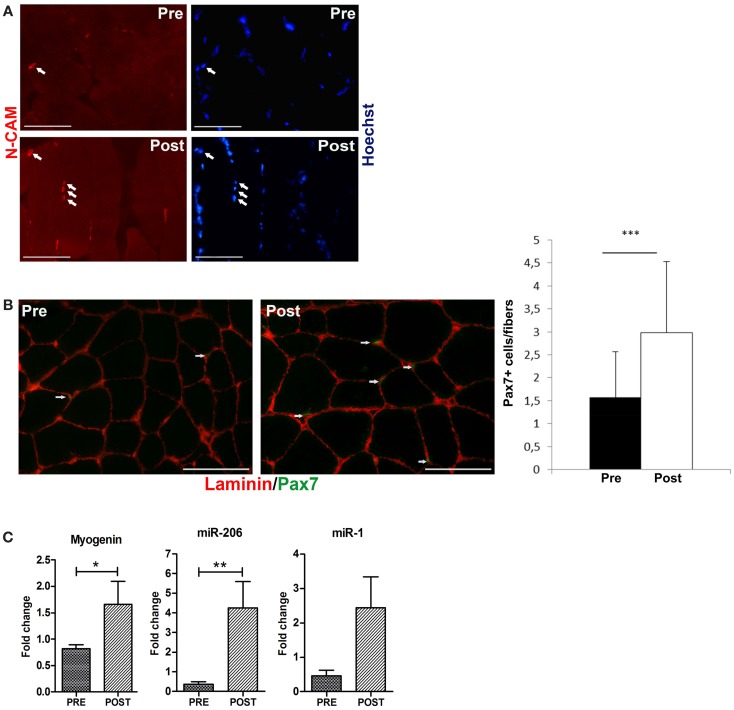
**Electrical stimulation induces an increase of satellite cells**. **(A)** Representative immunofluorescence analysis for N-CAM expression (red stained, arrowed). N-CAM expressing cells are increased in post-trained muscle compared with the pre-training condition. Nuclei are counterstained in blue with Hoechst. Bar 100 μm. **(B)** Representative co-immunofluorescence analyses of laminin (red staining) and Pax7 (green staining) expression in skeletal muscle biopsies comparing pre- to post-training conditions. The number of Pax7 positive cells (arrowed) is increased in biopsies of post-trained subjects, compared to the pre-training ones. Bar 100 μm. Right panel: percentage of Pax7+ cells in pre-trained and post-ES-trained muscles. Data are represented as average ± SD. ***p < 0.0001. **(C)** Real time PCR analysis for myogenin, miR-206, and miR-1 expression in pre-trained (PRE) and post-ES-trained (POST) muscles. Data are represented as average ± SEM. *n* = 16. *p < 0.05; **p < 0.005.

To verify whether ES promotes a similar response of exercise, we analyzed the expression of relevant molecular markers of activated and committed satellite cells such as N-CAM, Pax7, and myogenin (Carosio et al., 2011). Immunofluorescence analysis revealed that ES induced a significant increase in the percentage of N-CAM (Figure 2A) and Pax7 (Figure 2B left and right panels) expressing cells, along with a significant increase in myogenin expression, analyzed by RT-PCR analysis (Figure 2C). Recent studies have shown that muscle cell proliferation and differentiation are mediated by a collection of muscle-specific miRNAs (van Rooij et al., 2008). miR-206 is expressed in early phases of differentiation, whereas the expression of miR-1 is a marker of terminal differentiation and controls the expression of relevant enzymes in the response to oxidative stress (Chen et al., 2006; Rao et al., 2006; Cacchiarelli et al., 2010). Real time PCR analysis (Figure 2C) revealed a significant up-regulation of miR-206 and an increase of miR-1 expression in ES stimulated muscle compared to control muscle.

### Characterization of molecular pathways involved in ES-mediated muscle adaptation

To determine the adaptation changes of gene expression due to ES, we performed RT-PCR to quantify shifts in mRNA levels of a selected panel of genes involved in muscle growth and plasticity, in pre-trained (used as control) and electrical stimulated (treated) aged muscles. One of the key factors involved in skeletal muscle adaptations and growth is insulin-like growth factor-1 (IGF-1) (Musarò et al., 2001; Berg and Bang, 2004; Adamo and Farrar, 2006; Scicchitano et al., 2009; Kern et al., 2011).

**Figure 3:**
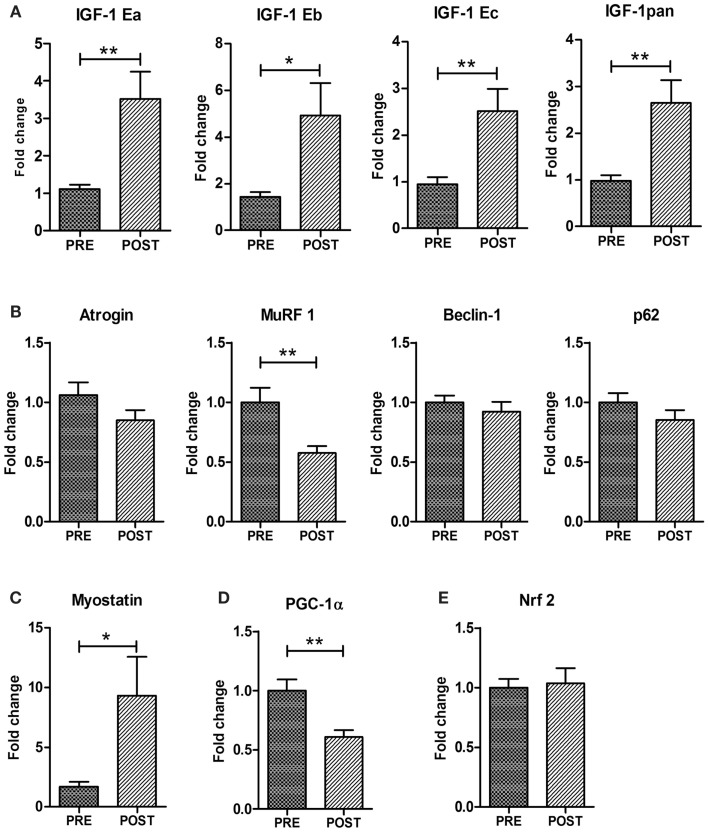
**Expression analyses of genes controlling muscle mass and metabolism**. Real time PCR analysis for the expression of IGF-1 isoforms (total IGF-1pan, IGF-1Ea, IGF-1Eb, IGF-1Ec) **(A)** Atrogin-1, MurF-1, Beclin1, p62 **(B)**, Myostatin **(C)**, PGC1α **(D)**, and Nrf2 **(E)**. Data are represented as average ± SEM. *n* = 16. **p < 0.005; ***p < 0.0005.

We analyzed the expression of the different isoforms of IGF-1. In humans, three mRNA variants (known as IGF-1Ea, IGF-1Eb, and IGF-1Ec) with alternatively spliced ends have been identified (Scicchitano et al., 2009; Vinciguerra et al., 2010). Figure 3A shows that ES promoted a significant increase in the mRNA expression of total (pan) IGF-1 and of IGF-1Ea, IGF-1Eb, and IGF-1Ec isoforms.

To verify whether ES stimulates not only anabolic pathways, but negatively modulates muscle catabolism, we analyzed the expression of factors associated with relevant proteolytic systems such as the ubiquitin-proteasome and the autophagy-lysosome systems (Vinciguerra et al., 2010). Atrogin-1 and MuRF-1 are muscle-specific atrophy-related ubiquitin ligases and are responsible for the increased protein degradation through the ubiquitin-proteasome system (Vinciguerra et al., 2010). We found a significant down-regulation of MuRF-1 and a reduced trend in atrogin-1 expression in the post-training group (Figure 3B). The autophagy-related genes Beclin1, Bnip3, and p62 did not change in trained muscles indicating that ES do not modulate the autophagy pathway (Figure 3B).

Another key modulator of muscle mass is myostatin (Elliott et al., 2012). Myostatin has been described as a negative regulator of skeletal muscle mass and regeneration and a target of miR-206 (Clop et al., 2006). Surprisingly, real time PCR revealed an up-regulation of myostatin mRNA in ES-treated muscle compared to control pre-trained muscle (Figure 3C).

The age-related decrease in muscle mass involves a selective loss of fast glycolytic fibers (Type II) over slow oxidative fibers (Type I) (Alnaqeeb and Goldspink, 1987). Although the Type I fibers are energetically more efficient than Type II fibers, so that senescent muscle should become progressively more resistant to fatigue, they are also greatly decreased in their force-generating capacity, exhibiting restricted contractile options in terms of speed and power output. Since ES involved an improvement in muscle strength and power, we analyzed one of the key factors involved in oxidative metabolism and fiber-type switching, namely PGC1α (Lin et al., 2002). Of note, PGC1α was down-regulated in ES-treated muscles (Figure 3D), indicating a maintenance of the fastest more powerful phenotype. Since down-regulation of PGC1α could point to altered mitochondrial function and therefore to potential increase in ROS production, we monitored the expression of Nrf2, a transcription factor that has a central role in oxidative stress response in worms, flies, and mice. Importantly, expression of the gene Nrf2 did not change with ES (Figure 3E).

Interestingly, muscle extracellular matrix (ECM) constitutes a vital adaptation in providing protection against contraction-induced injury in human skeletal muscle (Mackey et al., 2011).

**Figure 4:**
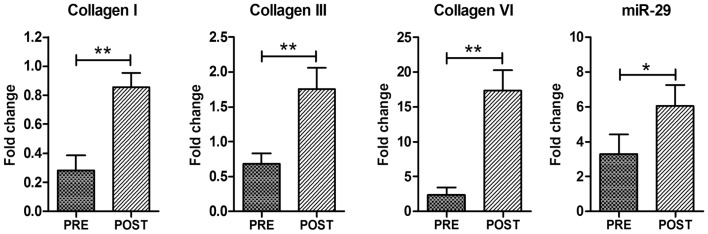
**Electrical stimulation promotes ECM remodeling**. Real time PCR analysis for Collagen I, Collagen III, Collagen VI, and miR-29. Data are represented as average ± SEM. *n* = 16. *p < 0.05; ***p < 0.0005.

To support this hypothesis, we analyzed, by real time PCR, the expression of adhesion-promoting matrix components, demonstrating a significant up-regulation of collagen types I and III in ES muscle compared to control pre-trained muscle (Figure 4). Of note, ECM represents also a niche component of satellite cells. One of the matrix components that might play a role in maintaining satellite cell function is collagen VI (Urciuolo et al., 2013). Real time PCR analysis revealed a significant increase in Collagen VI expression in ES-trained muscle compared to pre-trained muscle (Figure 4).

Interestingly, the up-regulation of ECM regulators was not associated with accumulation of fibrotic tissue, as revealed by histological analysis (Figure 1), suggesting that ECM remodeling is indeed an important homeostatic event promoted by ES. To support this evidence, we analyzed the expression of miR-29, which controls the extracellular proteins and the fibrotic process (van Rooij et al., 2008; Cacchiarelli et al., 2010; He et al., 2013). qRT-PCR revealed that ES promotes a significant increase in miR-29 expression (Figure 4), suggesting that mir-29 controls fibrosis in ES stimulated muscle.

## Discussion

Electrical stimulation has been proved to be very effective in restoring muscle mass and function in denervated muscles (Kern et al., 2004, 2008, 2010; Carraro et al., 2005; Ashley et al., 2007; Maddocks et al., 2013).

The aim of the present study was to verify whether ES can be used to improve muscle function in elderly individuals. It is well documented that training and regular exercise can attenuate the pathological signs of sarcopenia, increasing muscle strength while decreasing fall risk. Nevertheless, certain pathologic conditions (e.g., sarcopenia, osteoarthritis, disuse associated atrophy, muscular dystrophies, trauma, and injuries) limit the ability to perform physical exercise. An alternative effective intervention to improve muscle recovery is ES.

We evaluated the functional performance of ES-trained subject and analyzed the molecular signature of ES-mediated effects on skeletal muscle. In our study, we did not include untreated controls but we compared the functional performance of same subjects before and after ES training.

The results collected here suggest that ES, similarly to physical exercise, attenuate the functional decline associated with aging, improving muscle strength and mass, maintaining the overall size of muscle fibers (decreasing during aging), activating satellite cell, and guaranteeing muscle adaptation. Thus, ES should be protective for sarcopenia.

All functional tests and maximal isometric torque showed significant improvements after 9 weeks of ES training, counteracting age-depended mobility ability, frailty, risk of falling, while improving functional performance and ADL. Of note, the ES-treated subjected performed similarly to a 6-week resistance trained older adults (70.6 ± 6.1 years) (Fragala et al., 2014), assessed with TUGT, 5× Chair Rise and gait time.

It has been reported that female subjects displayed higher sensory and supramotor excitability to surface ES (Maffiuletti et al., 2008). In our study, the intensity (current) was very similar in all (male and female) subjects, which were instructed to increase the stimulation intensity until their maximum sensory tolerance level was reached. None of them declared problematic events during training sessions, and both males and females reported slight pain clinically not relevant at rest before ES, without changes through ES training. In the functional tests, the males were generally stronger and faster than females except chair rise (females faster) and stair test (equal). Both males and females improved in all tests but females gained in percentage nearly twice as males. This could be explained taking into consideration that the electrodes we used in our study covered a relative larger area of the thigh in female than male and therefore activated more motorpoints by stimulation. This results in training of a larger volume of the quadriceps muscles similar to the observation reported by Maffiuletti et al. (2008). We suggest, for all therapeutic applications of ES that aim to improve force and quality of muscle structure, to use electrode sizes greater than 100 cm^2^ to cover larger portions of the muscle and at the same time to create less discomfort due to low current density at the electrode-skin interface (Kern, 2014).

At molecular level, we demonstrated that ES promotes the modulation of factors associated with muscle growth and induces a remodeling of ECM. Our findings demonstrated that ES of 73.1 years old healthy sedentary seniors, increases expression of IGF-1 and of relevant biomarkers of activated satellite cells and myoblasts, reduces expression of muscle-specific atrophy-related ubiquitin ligase genes, and promotes the remodeling of myofibers and of ECM.

IGF-1 plays an important anabolic role in skeletal muscle and it is an important modulator of muscle growth and regeneration. Different evidences indicate that, during muscle regeneration, IGF-IEb levels is responsible for activating and for proliferating satellite cells; IGF-1Ea is responsible for myoblast differentiation and IGF-1Ec expression is normally up-regulated in response to mechanical signals (Matheny et al., 2010). Thus, our data suggests that ES stimulates the expression of different isoforms of IGF-1 in muscle, guaranteeing muscle homeostasis and protection against age-related sarcopenia. In fact, increased levels of IGF-1 were associated with a reduced level of expression of MuRF-1, a gene involved in muscle atrophy.

Among potential molecular mechanisms activated by exercise, autophagy might play a critical role for metabolic adaptation (Lira et al., 2013; Ferraro et al., 2014; Vainshtein et al., 2014). In particular, it has been reported that autophagy is an essential process for skeletal muscle adaptation and physical performance after endurance training (Lira et al., 2013). Conversely, we did not observe significant activation of autophagic pathway in ES-treated subjects. This can be justified considering that the up-regulation of autophagic-relevant markers is an early event and then they returned to basal levels shortly after the stimulus. We analyzed the muscle biopsies 7 days after the last ES treatments, a time point that might not sustain the activation of autophagic pathways. Further analysis will clarify and address this point.

Of note, myostatin was up-regulated in ES-treated muscles. We can interpret this result considering that myostatin may be produced locally by skeletal muscle cells to limit the muscle growth stimulated by IGF-1, guaranteeing an appropriate organ size (Shyu et al., 2005).

The up-regulation of myostatin can be also explained considering that ES guarantees a balance between satellite cells activation and differentiation. In fact, skeletal muscle differentiation is a complex and highly regulated process characterized by morphological changes, which include myoblast proliferation, alignment, elongation, and fusion into multinucleated myotubes. This is a balanced process dynamically coordinated by positive and negative signals. Recent studies revealed that IGF-1 also stimulates the expression of myostatin and it has been suggested that myostatin and IGF-1 positively coordinate myogenesis (Kurokawa et al., 2009; Valdés et al., 2013). Interestingly, it has been recently reported that myostatin stimulates C2C12 proliferation, and this effect occurred in the presence of IGF-1 (Rodgers et al., 2014). Thus, it is possible that in our experimental model the modulation of myostatin is independent by miR-206 expression/activity; however, myostatin and the relevant markers of activated and differentiating satellite cells are part of the mechanism for muscle adaptation induced by ES.

Interestingly, the up-regulation of collagen VI and ECM remodeling suggests that ES strengths key component of the satellite cell niche (Urciuolo et al., 2013). It has been recently suggested that ES stimulates satellite cells and a strengthened ECM, factors that are likely to be involved in protecting the muscle from damage on exposure to subsequent injuring stimuli (Mackey et al., 2011).

These results are also in agreement with morphometric analyses, which showed an increase of the percentage and diameter of the fast-type fibers.

Of clinical interest was the up-regulation of miR-29, which control fibrosis in different tissues, including skeletal muscle (Cacchiarelli et al., 2010). Considering that sarcopenia involves a decrease in muscle integrity as fibrotic invasions replace functional contractile tissue, and a progressive loss of the most powerful fast fibers, our data clearly indicate that ES improves muscle function and mass and protects against accumulation of fibrosis, regulating key factors, and signaling of muscle homeostasis and growth.

Altogether, the molecular data support our clinical findings that neuromuscular ES positively influences excitability and recruitment of stimulated muscle fibers resulting in greater force and better coordination guaranteeing, ADL, exercise programs, and rehabilitation strategies.

In conclusion, a three times a week ES is an effective therapy to improve molecular adaptations of muscle, counteracting muscle atrophy, and improving functional outcomes with positive influence on quality of life of seniors.

## Author Contributions

Helmut Kern, Samantha Burggraf, Nejc Sarabon, Matthias Krenn, Jan Cvecka: designed the clinical work, recruited senior subjects and made clinical evaluation, drafted the work, reviewed the work. Helmut Kern, Stefan Löfler, Michael Vogelauer, Winfried Mayr, Hannah Fruhmann: collected human biopsies and samples; designed the ES protocol, performed functional evaluation on senior subjects, acquisition, statistical analysis, and interpretation of clinical data, drafted the work, reviewed the work. Laura Barberi, Simona Sbardella, Vanina Romanello, Marco Sandri, Antonio Musaro: perform molecular analysis, acquisition, statistic analysis, and interpretation of gene expression data, reviewed the work. Laura Pietrangelo, Feliciano Protasi: performed electron microscopy analysis, measured frequency, and position of CRUs and mitochondria, acquisition, statistical analysis, and interpretation of data, drafted the work, reviewed the work. Sandra Zampieri, Ugo Carraro: performed histological analysis, acquisition, statistic analysis, and interpretation of data; drafted the work, reviewed the work. Antonio Musaro, Marco Sandri, Helmut Kern, Ugo Carraro, Feliciano Protasi, Sandra Zampieri: designed and organized the experiments, interpreted the results, critically revised the work. Antonio Musaro: wrote the paper. All authors approved the final version of the manuscript.

## Conflict of Interest Statement

The review process was handled objectively despite Luciano Merlini having collaborated with the authors. The authors declare that the research was conducted in the absence of any commercial or financial relationships that could be construed as a potential conflict of interest.
